# “Standing on common ground” - a qualitative study of self-management support for patients with multimorbidity in primary health care

**DOI:** 10.1186/s12875-020-01290-y

**Published:** 2020-11-17

**Authors:** Joel Freilich, Gunnar H. Nilsson, Mirjam Ekstedt, Maria Flink

**Affiliations:** 1 Department of Learning, Informatics, Management and Ethics, Karolinska Institutet, 17177 Stockholm, Sweden; 2 Department of Neurobiology, Care Sciences and Society (NVS), Karolinska Institutet, Stockholm, Sweden; 3 Department of Health and Caring Sciences, Linnaeus University, Kalmar/Växjö, Sweden; 4 Department of Social work in healthcare, Karolinska University Hospital, Stockholm, Sweden

**Keywords:** Primary health care, Multimorbidity, Self-management, Patient centered care, Telemedicine

## Abstract

**Background:**

Multimorbidity, the co-existence of two or more chronic conditions in an individual, is present in most patients over 65 years. Primary health care (PHC) is uniquely positioned to provide the holistic and continual care recommended for this group of patients, including support for self-management. The aim of this study was to explore professionals’, patients’, and family caregivers’ perspectives on how PHC professionals should support self-management in patients with multimorbidity. This study also includes experiences of using telemedicine to support self-management.

**Methods:**

A mixed qualitative method was used to explore regular self-management support and telemedicine as a tool to support self-management. A total of 42 participants (20 physicians, 3 registered nurses, 12 patients, and 7 family caregivers) were interviewed using focus group interviews (PHC professionals), pair interviews (patients and family caregivers), and individual interviews (registered nurses, patients, and family caregivers). The study was performed in urban areas in central Sweden and rural areas in southern Sweden between April 2018 and October 2019. Data were analyzed using content analysis.

**Results:**

The main theme that emerged was “Standing on common ground enables individualized support.” To achieve such support, professionals needed to understand their own views on who bears the primary responsibility for patients’ self-management, as well as patients’ self-management abilities, needs, and perspectives. Personal continuity and trustful relationships facilitated this understanding. The findings also indicated that professionals should be accessible for patients with multimorbidity, function as knowledge translators (help patients understand their symptoms and how the symptoms correlated with diseases), and coordinate between levels of care. Telemedicine supported continual monitoring and facilitated patient access to PHC professionals.

**Conclusion:**

Through personal continuity and patient-centered consultations, professionals could collaborate with patients to individualize self-management support. For some patients, this means that PHC professionals are in control and monitor symptoms. For others, PHC professionals play a less controlling role, empowering patients’ self-management. Development and improvement of eHealth tools for patients with multimorbidity should focus on improving the ability to set mutual goals, strengthening patients’ inner motivation, and including multiple caregivers to enhance information-sharing and care coordination.

**Supplementary Information:**

**Supplementary information** accompanies this paper at 10.1186/s12875-020-01290-y.

## Background

Multimorbidity is most commonly defined as the co-existence of two or more chronic conditions in an individual [1]. It is present in the majority of patients older than 65 years, increases with age [2], and is associated with decreased quality of life, functional decline, and increased health care use [3]. In Sweden, people with multimorbidity account for 50% of total health care costs [4].

Although multimorbidity is becoming increasingly common, health care is still largely organized to manage single diseases [5–7]. In Sweden, recent and proposed changes in legislation have emphasized that primary health care (PHC) needs to take the lead in coordinating care for people with multimorbidity [8, 9]. PHC is uniquely positioned in health care to provide the holistic and coordinated care recommended for this group of patients [3, 7, 10].

PHC in Sweden includes all care that does not require the resources of specialist health care or hospitals [11]. Thus, PHC involves PHC centers, rehabilitation and home health care, and a range of professionals, including physicians (mostly general practitioners [GPs]), registered nurses (RNs), assistant nurses, psychotherapists, physiotherapists, occupational therapists, social workers, and other allied health professionals. Structurally, the different units can belong to the region or the municipality, or they can be privately owned and operated under a contract.

Most of the time, patients with multimorbidity are expected to perform daily care activities and manage complex regimens on their own or with the support of family caregivers (FCs) [4]. It is therefore important for PHC to have strategies to support patients’ self-management, defined as “an individual’s ability to manage the symptoms, treatment, physical and psychosocial consequences and lifestyle changes inherent in living with a chronic condition” [12].

Living with multimorbidity often implies a range of challenges. Symptoms of pain, fatigue, and depression are common barriers to self-management [13, 14], and age-related changes in many people with multimorbidity may impair the functional ability necessary for proper self-management [15]. Chronic conditions also change over time, and patients need to change their priorities as different conditions dominate [16]. They also need to cope with a fragmented health care system and often with confusing and contradictory information from different health care professionals [13, 14]. Polypharmacy and an overwhelming treatment and self-management burden can result if physicians follow individual guidelines for each long-term condition experienced by a patient with multimorbidity [6, 15, 17].

Both professionals and patients experience patients’ engagement in self-management as a balance between the triangle of patients’ resources, patients’ motivation, and shared responsibility between professionals and patients [18]. To succeed, self-management should be patient-centered, i.e. support should be aligned with patients’ abilities, strengths, and value systems, and should respond to changes in their clinical conditions [16, 17, 19, 20]. Patient–professional relationships and communication are central to self-management support, which should be characterized by shared goals and shared understanding [21, 22].

There is still a lack of evidence that self-management interventions improve clinical outcomes and quality of life for patients with multimorbidity [23, 24]. However, interventions that target particular risk factors common to comorbid conditions or generic functional difficulties experienced by patients seem promising, as does a multidisciplinary team approach to self-management support [23]. Research on socioeconomically deprived populations indicates that longer consultation times may promote cost-effective self-management support, at least in such populations [25].

By addressing the holistic needs of patients with multimorbidity, eHealth (i.e. information and communication technologies used to facilitate health promotion and care) has the potential to help promote patient-centered care, and thus, self-management [26, 27]. However, both patients and professionals have been reluctant to adopt eHealth tools [28–31]. Research shows that eHealth interventions are a cost-effective way to support self-management of single diseases [32], but evidence about multimorbidity is lacking.

Thus, more evidence is needed about the best ways for PHC to provide self-management support for people with multimorbidity. Whereas most studies on self-management support have focused on either patients’ or professionals’ experiences, this study covers the experiences of professionals, patients, and FCs. In this study, the professionals include physicians and RNs, the largest groups of professionals in PHC. The study also includes experiences of using telemedicine (i.e. remote clinical services) to support self-management.

The aim of this study was thus to explore professionals’, patients’, and family caregivers’ perspectives on how PHC professionals should support self-management in patients with multimorbidity.

## Methods

### Design

Because the study included two sets of data, we deemed a qualitative mixed methods design appropriate [33]. Focus group and in-depth interviews with professionals and patients in central Sweden comprised the core data. Complementary data came from in-depth interviews with RNs, patients, and FCs from a rural region in southern Sweden that uses telemedicine to support patients with chronic heart failure. These data were included to obtain maximum contextual variation and to provide information about telemedicine as a tool to support self-management [34].

### Setting and sample

**Fig. 1:**
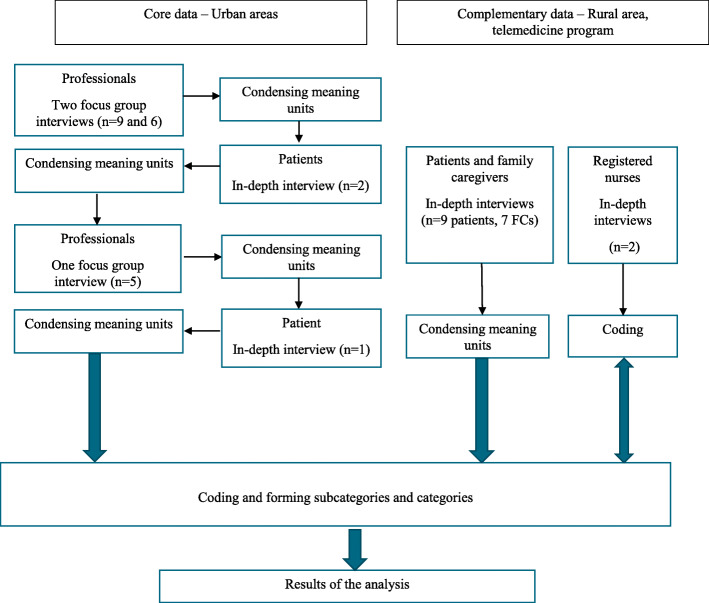
The (mixed methods) collection and analysis process

The study was carried out between April 2018 and October 2019 in urban areas in central Sweden, and in a rural area in southern Sweden (Fig. 1). Whereas two of the urban areas are located in suburbs of Stockholm that are close to university hospitals, the third is located in a municipality with a population of around 60,000 that has its own hospital. The rural area in southern Sweden has around 10,000 inhabitants and is situated 40 km from the nearest hospital.

A total of 42 participants, including 20 physicians (14 women), 3 RNs (all women), 12 patients (6 women) and 7 FCs (all women), were interviewed in the study (Table 1A and B).

**Table 1:** Demographics of patients and health care professionals

A: Demographics of patients			
**Patient**	**Age**	**Gender**	**Civil state**
1	69	Woman	Single
2	82	Woman	Widow
3	71	Man	Common-law husband
4	86	Woman	Widow
5	78	Man	Married
6	91	Woman	Widow
7	64	Man	Married
8	96	Woman	Widow
9	71	Man	Married
10	86	Man	Married
11	87	Man	Married
12	85	Woman	Common-law wife
B: Demographics of health care professionals			
**Phycisian**	**Age**	**Gender**	**Working years as professionals**
1	48	Woman	4
2	40	Man	0
3	37	Woman	Resident physican
4	55	Woman	4
5	65	Woman	28
6	55	Woman	15
7	30	Man	Resident physican
8	38	Woman	4
9	62	Woman	24
10	37	Man	2
11	61	Woman	24
12	43	Woman	Resident physican
13	64	Woman	14
14	36	Man	0
15	36	Woman	0
16	38	Man	0
17	27	Woman	Medical doctor
18	43	Woman	10
19	36	Man	Intern
20	55	Woman	10
**Registred nurse**			
1	60	Woman	19
2	56	Woman	20
3	37	Woman	6

#### Health care professionals

All physicians and one RN were recruited from three PHC centers in the urban areas in central Sweden. Two RNs were recruited from a PHC center in the rural area of southern Sweden. One was a coordinator for the telemedicine program, and the other worked in the municipality’s home care unit. We chose PHC centers that had earlier shown an interest in participating in research and that had a stable staff situation. Fifteen of the physicians were GPs, and the remaining five were physicians-in-training. The physicians’ mean age was 45 years, and the GPs had worked in their profession for a mean of 13 years. The RNs were a mean of 51 years and had worked in their profession for a mean of 15 years.

#### Patients from the urban areas

Three patients came from urban areas in central Sweden (patients 1–3, Table 1). They had been hospitalized for chronic heart failure or chronic obstructive pulmonary disease (COPD), had been part of the control group in an RCT study [35], and had agreed to participate in a qualitative evaluation of their care. To be included in this study, they had to have at least one additional chronic disease in a different organ system. People with cognitive impairment were excluded from the study. All patients had access to home care when needed.

#### Patients and family caregivers from the rural area

Nine patients (patients 4–12, Table 1), and seven FCs were recruited from the PHC center in southern Sweden. The patients were involved in the telemedicine program that was part of a regional effort to support patient-centered and seamless care for older patients. The telemedicine program targeted patients with chronic heart failure and/or diabetes, and included registration of health parameters in a tablet computer (blood pressure, weight, temperature, oxygen saturation, and - for those with diabetes - blood sugar). Patients also filled out questions about their health condition and symptoms related to chronic heart failure. Two RNs at the PHC center monitored patients’ registrations daily. The RNs could react to changes in patients’ conditions by contacting them, and by involving their PHC physician or home care nurse. The patients could also use the tablet for video meetings with the RNs. The frequency of registrations and contact with the RNs was decided individually by the patient and the RN. No goal setting or motivational support was provided as part of the telemedicine program. Patients received supportive care from RNs in the home care unit when needed, but they were not directly involved in the telemedicine program. The inclusion and exclusion criteria were the same as for the patients in urban areas. All the FCs except one (a daughter) lived with the patient.

The mean age of all participating patients was 80 years. The most frequent diseases were chronic heart failure, COPD, arthritis, and diabetes.

### Data collection

The PHC centers were recruited by JF, who provided the participating professionals with oral and written information about the study. Patients from urban areas were informed about the study via telephone by JF. Patients and FCs from rural areas were invited by one of the two RNs who coordinated the telemedicine program; the RN gave them oral information about the study.

Data were collected sequentially (Fig. 1). The research group developed an initial interview guide for the focus group interviews with professionals. Questions were open-ended and about 1) how professionals perceived their role in supporting self-management for patients with multimorbidity, and 2) how patient-centered the professionals perceived this support. It was important to include questions about patient-centeredness because PHC professionals are trained in patient-centered care, and earlier studies suggested that such care is central to successful self-management support [16, 17, 19–22]. Questions covered communication (e.g, “How do you usually inform patients about self-management, for example, medications, tests, smoking, and so on?”) motivational work (e.g. “How do you get information about the patient’s level of motivation?”), coordination of care (e.g. “How do you view your role as a coordinator in health care?”), continuity of care (e.g. “How do you view personal continuity in contrast to reading information about the patient in the medical chart?”), shared information (“How do you view the situation that patients have access to their medical information?”) and shared decision-making (e.g. “What is your view of setting health goals together with your patient?”). The research group also developed a patient interview guide with corresponding open-ended questions about what support the patients wished for from PHC. Between the interviews, the researchers wrote and discussed memos to develop appropriate new questions that were added to the interview guide for the following interviews. For instance, early interviews made it clear that patients and professionals interpreted the term “self-management” differently. The following interviews therefore started with an open-ended question about what self-management meant to the participants. In the first focus group with professionals, participants brought up eHealth as a tool to support self-management. They did not, however, have a clear idea how eHealth could be used to support patients with multimorbidity. This resulted in the decision to include a group of patients with multimorbidity who had experience with telemedicine, the subgroup from the PHC center in southern Sweden.

We chose to use focus groups with professionals for practical reasons: it is challenging to schedule individual interviews with professionals, as they are often pressed for time. Focus group discussions also have qualities of both interviews and discussions and benefit from group dynamics because they stimulate participants to react to, reject, or confirm statements from other participants [36]. The focus groups were led by two of three researchers (JF, MF, or ME). One moderated the discussion and the other ensured that all topics were covered. The focus groups consisted of nine, six, and five professionals. Participants were asked to keep in mind patients with multimorbidity, whose diseases, including mental disorders, had a major impact on their everyday lives. They were also asked to think of diseases and treatments that were complex for patients and professionals to evaluate because of polypharmacy or overlapping symptoms (e.g. shortness of breath in COPD and chronic heart failure).

We also chose to conduct in-depth interviews with patients and FCs, for practical reasons. It was easier to interview patients in their homes because their health conditions often made it difficult for them to participate in group interviews. There were too few RNs to form a focus group. Interviews with patients and FCs were performed by JF and two research assistants. Five of the interviews were with a patient and his or her FC; that is, two people were interviewed at the same time. The two final RN interviews were performed via telephone by JF.

Focus group interviews lasted for 40 to 65 min, and individual interviews lasted for 20 to 45 min. All were audiotaped and transcribed verbatim.

### Analysis

We used an inductive approach to content analysis which was deemed appropriate to inductively explore a perspective that is not well-explored [37]. After each interview, the transcript was read and analyzed several times by JF, MF and ME. The analyses comprised descriptions of the manifest content, capturing the visible or obvious content close to the text. Analyses also comprised descriptions of the latent content, capturing the underlying meaning of the content, distant from the text but still close to the participants’ lived experiences [38]. Then the text about the topic of the study was divided into meaning units that were condensed. After all interviews, the condensed meaning units were abstracted and labeled with a code. Next, the research group met to compare and organize the condensed meaning units into categories and subcategories [37, 39]. The categories and subcategories were then presented to and discussed with two groups of researchers not involved in the study. Two final interviews were then conducted to check whether any new data emerged; these were the telephone interviews with the two RNs from southern Sweden. These interviews did not result in any new categories. A main theme emerged from the categories as a result of analyzing latent findings in the data.

## Results

The main theme was “Standing on common ground enables individualized support.” This theme was supported by four categories: 1) Individualized support and patient-professional relationships, 2) Professionals as knowledge translators to help patients learn self-management skills, 3) Managing and coordinating multimorbidity in a system focused on single diseases, and 4) Shifting roles and differing views of responsibility for self-management. Categories and subcategories are presented in Table 2, and key words in the subcategories are italicized in the text.

**Table 2:** Categories and subcategories

Categories	Subcategories
**Individualized support and patient-professional relationships**	Individualized care considers the patient’s agenda and self-management ability
	Trustful relationships enable self-management support
	Support for self-management is more than information
**Professionals as knowledge translators to help patients learn self-management skills**	Knowledge affects self-management abilities and decreases anxiety
	Pedagogical strengths and weaknesses among health care professionals
	Self-monitoring enables self-management
**Managing multimorbidity and coordinating care in a system focused on single diseases**	PHC’s role in coordinating care in a fragmented system
	Accessibility to health care and time for patients with multimorbidity enhances self-management support
**Shifting roles and differing views of responsibility for self-management**	When PHC professionals are in control, it increases adherence and patient safety and reduces anxiety
	Empowering patients enables a shift in responsibility

Abbreviation: *PHC* primary health care

### Individualized support and patient-professional relationships

Patients and professionals said that health care should be *individualized* by taking the patients’ agendas into account and considering their knowledge about and capacity for self-management. Although patients had been invited for follow-up PHC visits about their chronic conditions, they were not always interested in talking about them, but rather could prefer to discuss symptoms or other concerns, such as a new skin lesion or hip pain. Professionals emphasized the importance of answering patients’ questions before bringing up their own agenda for the visits. They noted that if they did not answer patients’ questions, the patients sought care at other places or trusted what they read in newspapers or what neighbors said more than health care guidelines. Several of the patients brought up examples of feeling disappointed when their questions were not answered. One said, “I’m worried about my leg that becomes numb. Instead of investigating it, they gave me a walker and transportation service, but that doesn’t solve the problem” (Patient 1).

Professionals and patients brought up the importance of patients’ individual goals. When discussing goal-setting, physicians and RNs mostly thought of clinical goals such as blood pressure and blood sugar. Patients, on the other hand, talked about symptom relief and health goals related to things they appreciated in their everyday lives, such as working in the garden or being with their grandchildren. One patient was clear about not wanting preventive drugs, only those that gave symptom relief: “My goal is not to become 100, but to have pain relief, nothing else” (Patient 2).

Participants said that a *trustful relationship* facilitated self-management support. Patients and FCs appreciated to encounter professionals they knew and trusted. Even though a new professional could have read the information in the medical record, they preferred to see someone they had known for a long time. They thought that this person knew them, which meant they did not have to repeat their medical history and that the person cared about them. PHC physicians thought they had a better opportunity than hospital physicians to see their patients as individuals because they had known the patients for a longer time and were familiar with their family situations. This was something that RNs also mentioned as important. “Through home visits, I can get another picture of the person and the FC. It’s a whole image that you can’t achieve by reading the medical record” (RN 1).

Several things could negatively influence patient-professional relationships, such as a lack of trust in the professional’s competence. A couple of patients brought up not trusting their PHC physician’s competence in treating heart disease. Difficulty understanding a physician whose native language was not Swedish could also influence patient trust. One patient said: “And then there is this language problem that results in misunderstandings. It makes me feel so helpless” (Patient 1).

Physicians, patients, and FCs, talked about *support that went beyond information and disease management*. Physicians mentioned that patients often wanted to contact them because of anxiety, stress, or loneliness, not primarily because of disease. Patients brought up loneliness as something frightening, and one patient had continued to participate in the telemedicine program not for health reasons, but because she wanted the social contact.

### Professionals as knowledge translators to help patients learn self-management skills

Physicians and patients reported that *knowledge and understanding of their diseases, symptoms, and treatments were important for both self-management abilities and reducing anxiety*. They thought that PHC had an important role to play as knowledge translator. Physicians saw it as their role to help patients understand their symptoms and how these symptoms correlated with their diseases. This was especially important when patients had several diseases that could cause the same symptoms (for example, COPD and chronic heart failure can both cause shortness of breath). “Patients know their symptoms, but why they have these symptoms and these problems and how they’re correlated with their disease, it’s our role to try to explain it to the patients” (Focus group 2).

Patients gave examples of how they had learned to act in response to different symptoms, e.g. increase their dose of medicine to decrease swelling in their feet. Patients also talked about when to initiate contact with the PHC center. For example, one patient would call her doctor if increasing the dose did not sufficiently decrease swelling. Patients brought up lack of knowledge and lack of ability using technology as barriers to self-management. Sometimes health problems (e.g., hearing impairment) made it hard for patients to understand new information; in these cases physicians needed the support of FCs.

Patients also gave examples of how knowledge and understanding could reduce anxiety. It was more frightening to experience symptoms that they could not interpret than to experience those that they understood. Some diseases, such as myocardial infarction, were more frightening than the chronic pain they had lived with for a long time, and those that were threatening made them more prone to make lifestyle changes.

Whereas patients talked about professionals’ *pedagogical failings*, professionals focused on their *pedagogical strengths*. Examples of pedagogical failings that patients mentioned included not answering patients’ questions and not explaining why different health parameters were to be checked (e.g. why patients needed to monitor their oxygen saturation). “Then I have this little tool that measures the oxygen level in my blood and that is new. I haven’t seen it elsewhere, and don’t really mind” (Patient 6). One patient who smoked also said that she was often met with admonitions that did not make her more likely to quit. As examples of pedagogical strengths, professionals noted the pedagogical tools they used in their consultations with patients. Physicians used analogies and pictures to explain concepts and sometimes printed out patients’ medicine lists and wrote clarifying information on them. To confirm that patients had understood them, physicians and RNs used questions that required the patients to summarize what the professional had said. One RN gave an example of how she could reassure herself that the patient had understand the physician’s prescription: “And then I ask about the blood pressure medicine, were you suppose to continue with it or not?... I try to have a conversation with the patient and see if they understood the information, and if they didn’t, I try to help them to understand.” (RN 3). The same RN also gave an example of a pedagogical failing neglegting to set goals for patients who feel unwell. “Normally I don’t set health goals with my patients if they’re too sick” (RN 3).

Physicians, patients, and FCs also talked about *self-monitoring* of health parameters. Patients sometimes initiated this monitoring on their own and sometimes at the request of professionals. Such monitoring could help patients better understand their diseases and thus facilitate self-management. For instance, patients felt they understood their blood pressure and blood sugar by measuring them, and in some cases, they learned how to respond to the results of the measures by changing medication regimens themselves. “Recording my weight has become like a compass. It helps me to adjust my medication” (Patient 10).

### Managing and coordinating multimorbidity in a system focused on single diseases

This category deals with problems related to a health care system that the professionals and patients both saw as focused on single diseases. This meant that PHC played an important *role in coordinating care in a fragmented system*. Fragmentation of care forced patients with multimorbidity to visit different specialists every year for their diseases. Physicians reported that younger patients could have the energy to come to many visits, but older patients often appreciated being able to consult the same physician about all of their health problems. “It becomes an involuntary full-time job for the patients; it’s their 40-hour week” (Focus group 3).

Care coordination could be a bothersome issue for patients with multimorbidity. Physicians, patients, and FCs all gave examples of such problems. Patients mentioned that they did not know whether their PHC physician and hospital specialist communicated or not, and they did not even know if the different professionals at the PHC communicated with each other. Home care patients and FCs who registered health parameters in the telemedicine program could only share this information with the PHC center, not with the nurses who provided in-home care. This was because the home care nurses were employed by the municipality, whereas the PHC center belonged to the region. Physicians not only thought that specialized care was fragmented, but also experienced fragmentation at their PHC centers, where RNs have become increasingly specialized in different areas, such as diabetes or COPD. One said, “Our RNs have become more and more specialized, while we as physicians are the only ones left who have the general picture” (Focus group 3).

According to physicians, existing electronic medical records were a technical barrier to coordinated care, as were health apps, both of which they described as disease-oriented.

On the other hand, there were examples of better care coordination. The RNs talked about their role as coordinators of different caregivers for patients with multimorbidity. FCs, home care providers, care managers, and allied health professionals often played a crucial role for patients with multimorbidity, and the RNs saw themselves as being the person who pulled it all together. Patients also gave examples of coordinated care. One FC appreciated that the cardiologist made visits to the PHC center: “They’re an incredible team, together with all of you who take care of my husband” (FC 1).

Most physicians also saw it as their role to coordinate care and keep medication to a reasonable level for their older patients with multimorbidity. Some regarded themselves as their patients’ advocate in dealings with other specialists. One physician said: “I’m the patient’s gladiator against the system” (Focus group 1).

*Accessibility to health care and time for patients with multimorbidity* was also important for supporting self-management. Physicians wished for more time with these patients so that they could clinically evaluate the patients’ complex health problems. Physicians explained that patients with multimorbidity were crowded out because of increased access to PHC visits for people with all sorts of health problems, even problems earlier regarded as manageable at home. This was the result of changes in regional health care policy and led to long waiting times that made some patients with multimorbidity accumulate health concerns they wanted to bring up. One physician said, “It’s hard to solve everything at one visit, it just becomes messy” (Focus group 2).

Patients also brought up the importance of easy accessibility to PHC. When one patient first started to participate in the telemedicine program for chronic heart failure, he worried that he would lose this accessibility. “From the beginning, I didn’t think I could meet with doctors or nurses,” he said. When asked about what self-management meant to him, he replied, “It’s when the doctors don’t have the time for us anymore” (Patient 5).

### Shifting roles and differing views of responsibility for self-management

This category describes the shifting roles that PHC professionals took to support patients’ self-management, as well as differing views of who is responsible for self-management.

A professional’s role in supporting self-management could shift by patient. Some patients with multimorbidity coordinated and managed their care independently, whereas others needed more support. Views of responsibility for self-management also differed, not just among professionals, but also among patients and FCs. Some preferred the professional to take a more controlling role; others emphasized the need for the professional to let go of control and to empower patients.

Professionals, patients, and FCs could believe that when *professionals were in control*, patients adhered to treatment better and felt safer and less anxious. One physician who believed it was important for her to maintain control said that she had to schedule regular checkups for patients to prevent their health from deteriorating. Otherwise, she thought they would not follow her advice. “I assume no one does what I say,” she stated (Focus group 2). Personal continuity could help physicians feel in control because it was easier to see changes in the clinical status of patients they had met before. Patients appreciated it when professionals had control of patient-related information, both through familiarity with a patient’s medical history and through ongoing monitoring. Several patients in the telemedicine program felt secure knowing that someone was keeping track of their health parameters and that the nurse would contact them if there was a change. This knowledge even prevented some from going to the emergency department. “I feel very secure,” said one patient. “Before, when I got chest pain or was anxious, I considered calling the ambulance. Now I don’t need to anymore” (Patient 8).

FCs could also play an important part in helping professionals see changes in the patients’ conditions: “I notice changes in my husband’s condition by being there every day,” said one person (FC1). For patients with more severe disability, support from a FC was not enough, and they needed extra assistance from the home care unit.

Some professionals regarded it as their role to motivate patients to take more responsibility for self-management. They favored *patient empowerment* to help patients take more responsibility for self-management, and believed that with the right knowledge, patients could manage their diseases better. The professionals were more likely to hand responsibility over to patients after having made sure the patients had such knowledge. “We want them to at least have the knowledge to make them live in symbiosis with their diseases in some way, and not let the disease become a monster that someone else takes care of” (Focus group 1).

Professionals also described how patients who managed their diseases more independently gave the professionals valuable time to see other patients in need. For most patients and FCs in the telemedicine program, recording the information felt meaningful and became an important daily routine. For others, this shift in responsibility felt challenging at times, and some patients were so sick that their FC had to record the information on their behalf.

Professionals also brought up personal continuity as an important tool to motivate patients in the long term. One said, “If you want to achieve a goal, it’s much easier to follow up your patient on a regular basis than to say, ‘See you in half a year’” (Focus group 1).

Professionals could find it challenging to motivate patients to take more responsibility, but it was easier with patients who already had a degree of motivation than those who did not. Physicians promoted group activities for patients and collaborated with RNs at the PHC center to talk about lifestyle changes with the patients. They also thought eHealth tools could play a role in this motivational work but could find it challenging to motivate patients to continue using these tools. “It’s not as fun to brush your teeth with an old toothbrush as with a new one” (Focus group 1).

Whereas some patients preferred professionals to take a lot of responsibility for managing the patients’ diseases and coordinating their care, others preferred to maintain overall control. They contacted the PHC center when they felt they needed to, and some would also independently change their medication regimens in agreement with their GP. “Then I increase my cortisone dosage myself, with the knowledge of my physician,” said one patient (Patient 2).

## Discussion

This qualitative study explored the perspectives of PHC professionals, patients, and FCs about how professionals should best support self-management in people with multimorbidity. Professionals, patients, and FCs thought it was important for the professionals to be accessible and support patient self-management. This was enabled by personal continuity, which facilitated trustful relationships. According to participants, PHC professionals should also function as knowledge translators and should coordinate between levels of care. Two perspectives on responsibility emerged. Professionals, patients, and FCs could consider professionals responsible for managing patients’ diseases or could think that professionals should support patients in taking the lead in self-management. Latent in the findings was the desire for individual support for self-management, and the overall theme that emerged was that standing on common ground enables such support. In other words, to support patients’ self-management, the professionals must first understand their own perspectives on who is primarily responsible for self-management. They must also understand patients’ and FCs’ preferences, needs, and perspectives on self-management and seek common ground with them on the support they need and on the distribution of responsibility.

Professionals’, patients’, and FCs’ wishes for mutual agreement enabled by trustful relationships and personal continuity are congruent with recommendations on patient-centered care (PCC) for people with multimorbidity [24]. PCC “*(a)* explores the patients’ main reason for the visit, concerns, and need for information; *(b)* seeks an integrated understanding of the patients’ world—that is, their whole person, emotional needs, and life issues; *(c)* finds common ground on what the problem is and mutually agrees on management; *(d)* enhances prevention and health promotion; and *(e)* enhances the continuing relationship between the patient and the doctor” [40]. The continuing relationship, i.e. the personal continuity emerged as important to several aspects of support. It facilitated trustful relationships and familiarity with patients’ health histories. Professionals also viewed personal continuity as a tool to detect deterioration in patients’ conditions and motivate patients to make and maintain lifestyle changes. For patients with multimorbidity, personal continuity can also help coordinate and bring order to the chaos that can result from the single-disease orientation of the health care system [3]. Observational studies show that personal continuity in PHC is associated with greater care satisfaction and better adherence to medical and health-promotion advice, less frequent hospital visits, and even decreased mortality [41, 42].

Participants expressed a need for individualized support. Professionals wanted longer consultations to properly clinically evaluate and support patients whose symptoms and treatments were complex. This finding is in line with UK recommendations for patients with complex multimorbidity and can help improve quality of life for patients in deprived areas [3, 7, 25]. In our study, professionals and FCs brought up the importance of teams, especially in supporting patients with complex needs. This agrees with expert recommendations for treating complex multimorbidity [3, 7]. Professionals also suggested that patients with less complex needs whose diseases are under control could have shorter or less frequent visits, leaving more time for those with greater needs. Nurses or assistant nurses could follow-up patients whose diseases are under control.

In Sweden, medical students and resident physicians becoming GPs are trained in patient-centered consultation. They practice eliciting patients’ ideas, concerns, and expectations early in consultations [43]. Nevertheless, patients in our study experienced shortcomings in patient-centeredness; an example was neglecting to listen to patients’ concerns. Patients and professionals also had different views of goals. Whereas professionals generally had clinical goals, patients wished for better quality of life. This result is in line with those of an analysis of Swedish National Patient Survey data from the mid-2000s that showed deficiencies in involving PHC patients in planning their care [44]. A model for shared decision making, such as the “Ariadne principle,” might help close the gap between professionals’ clinical goals and patients’ individual goals [45]. In practice, this could mean asking patients at the beginning of the consultation about what is bothering them the most and what they would like to focus on. Similarly, a Cochrane report on managing chronic conditions brings up the importance of personalized care planning. The authors define this as a “collaborative process used in chronic condition management in which patients and clinicians identify and discuss problems caused by or related to the patient’s condition, and develop a plan for tackling these. In essence it is a conversation, or series of conversations, in which they jointly agree goals and actions for managing the patient’s condition” [46].

In our study, we found differing perspectives on responsibility. Some patients and professionals preferred PHC to take the overall responsibility for patient self-management, whereas others preferred letting patients take more responsibility. This finding is in line with those of a recently published study that concludes that patients with multimorbidity vary in their needs and preferences about being in charge of their care [47]. Our findings reflect an ongoing paradigm shift in chronic illness management. In the traditional view, professionals are experts and patients have little to bring to the table. In the newer paradigm of collaborative care, patients and physicians work in partnership [48]. Patients are the experts on their own lives, and physicians are the experts on diseases. However, we and others [49] have found that physicians can struggle with sharing or letting go of control. We also found that some patients struggle with taking control. Reasons could include lack of knowledge and functional and/or psychological impairment. We found that particularly patients with more severe disability had difficulties to cope with the self-management tasks they were asked to complete. This brings to the fore what earlier studies also found, that self-management interventions can be too demanding for this group of patients [6, 15, 17, 50]. For these patients, FCs played a particularly important role, supporting them during PHC center visits and surveilling their condition at home. Also, the home care unit played a crucial role for these patients. Further, we found that a telemedicine program could promote care access and surveillance for these patients. Trust in surveillance could be a double-edged sword. Patients reported feeling less need to go to the emergency room in response to symptoms, which is positive if the condition does not require emergency care (e.g. anxiety). However, it is negative if the patient disregards signs of an emergency, believing that the PHC center would tell them if they needed hospital care.

In collaborative care, professionals aim to inform patients and support their inner motivation in order to empower them and facilitate self-management [48]. We found examples of achieving these goals in our study. For example, patients who understood their symptoms could manage their diseases better. Self-monitoring sometimes facilitated learning, especially when supported by interpretations from the professional. It also became an important routine for many patients that they incorporated into their daily lives.

### Strengths and weaknesses

Participant selection had both strengths and limitations. In the interviews with professionals, we asked them to think of patients whose diseases and treatments had a major impact on their everyday lives and were complex for the professionals and patients to manage. We also made an effort to recruit patients with complex needs by inviting those who had recently been hospitalized or who PHC professionals thought needed self-monitoring and continual contact with the PHC center. However, some patients in the telemedicine program did not have diseases that were complex to manage but participated in the program for other reasons (e.g. increased sense of security). Additionally, our study excluded some of the frailest older adults, such as those with cognitive impairment and dementia.

The inclusion of professionals, patients, and FCs from both urban and rural areas increased data variation. Using a mixed qualitative design gave us the opportunity to gather data on telemedicine as a tool for supporting self-management. Such data are of particular relevance, since many people with multimorbidity have been reluctant to use eHealth [28, 29]. The Covid-19 pandemic has highlighted the value of functional strategies for telemedicine. Unfortunately, we did not have the opportunity to interview PHC physicians about their experiences of the telemedicine program.

The authors come from different disciplines (family medicine, nursing, social work, and health care research). This brought a multidisciplinary perspective to the research and helped ensure triangulation. JF and GN were able to call on their experience as clinically active GPs. ME and MF, on the other hand, were experienced in home health care delivery and in interviewing patients and professionals about care transitions. GN’s and ME’s academic experience of the organization of care delivery enriched the discussion section. Credibility was increased through peer-debriefing with two groups of researchers, one in health care implementation sciences, and one in multimorbidity.

### Implications for health care

The findings of this study imply a need for ongoing training in patient-centered communication and shared decision-making. It should also be a priority to develop guidelines for managing multimorbidity that focus on personal continuity, individualized consultation length, and multidisciplinary care. Development and improvement of eHealth tools for patients with multimorbidity should focus on tailoring the tools to the needs of both clinicians and patients. The tools should not only help control disease but also empower patients. In practical terms, this means improving the ability to set mutual goals, strengthen patients’ inner motivation, and include multiple caregivers to enhance information-sharing and care coordination.

## Conclusion

In this study, we found that PHC played an important role in supporting self-management for patients with multimorbidity. Through personal continuity and patient-centered consultations, professionals could collaborate with patients to individualize this support. For some patients, this means that PHC professionals are in control and monitor symptoms. For others, PHC professionals play a less controlling role, empowering patients’ self-management. Customized support requires a personal care plan that is revised as patients’ circumstances change. This plan should take into account clinical goals, patients’ abilities and knowledge, support from other caregivers, and above all, patients’ own goals, which often focus on improving quality of life.

Because it supports continual monitoring and easy access to PHC, telemedicine is a potential tool for supporting self-management, especially for patients with multimorbidity who are at risk of rapid somatic deterioration. It might also be useful for patients who want extra support, such as those who feel anxious or alone. We also found that self-monitoring helped some patients learn to understand their diseases and symptoms better and became an important part of their daily routine.

## Supplementary Information

**Additional file 1.** Examples of categories, subcategories, and codes/quotations color-coded by origin: black for physicians, green for RNs, blue for patients, and red for FCs.

## Data Availability

The datasets used during the current study are available from the corresponding author on reasonable request.

## Abbreviations

COPD: Chronic obstructive pulmonary disease
FC: Family caregivers
GP: General practitioner
PHC: Primary health care
PCC: Patient centered care
RN: Registered nurse

## References

[1] van den Akker M, Buntinx F, Metsemakers JF, Roos S, Knottnerus JA (1998). Multimorbidity in general practice: prevalence, incidence, and determinants of co-occurring chronic and recurrent diseases. J Clin Epidemiol.

[2] Barnett K, Mercer SW, Norbury M, Watt G, Wyke S, Guthrie B (2012). Epidemiology of multimorbidity and implications for health care, research, and medical education: a cross-sectional study. Lancet..

[3] Wallace E, Salisbury C, Guthrie B, Lewis C, Fahey T, Smith SM (2015). Managing patients with multimorbidity in primary care. BMJ..

[4] The Swedish Agency for Health and Care Services Analysis. VIP i vården? – Om utmaningar i vården av personer med kronisk sjukdom. Stockholm; 2014. https://www.vardanalys.se/rapporter/vip-i-varden/.

[5] Guthrie B, Payne K, Alderson P, McMurdo ME, Mercer SW. Adapting clinical guidelines to take account of multimorbidity. BMJ. 2012;345.. 10.1136/bmj.e6341 23036829

[6] Boyd CM, Darer J, Boult C, Fried LP, Boult L, Wu AW (2005). Clinical practice guidelines and quality of care for older patients with multiple comorbid diseases: implications for pay for performance. JAMA..

[7] Moffat K, Mercer SW (2015). Challenges of managing people with multimorbidity in today's healthcare systems. BMC Fam Pract.

[8] Lagen om samverkan vid utskrivning från sluten hälso- och sjukvård. (SFS 2018:612). Stockholm: Ministry of Health and Social Affairs.

[9] Ministry of Health and Social Affairs. God och nära vård - En primärvårdsreform. Delbetänkande av utredningen God och nära vård – Vård i samverkan (SOU 2019:29). Stockholm: Nergård, A.

[10] Roland M, Paddison C. Better management of patients with multimorbidity. BMJ.2013;346:f2510. 10.1136/bmj.f2510 23641032

[11] Hälso- och sjukvårdslagen. (2017:30). Stockholm: Ministry of Health and Social Affairs.

[12] Barlow J, Wright C, Sheasby J, Turner A, Hainsworth J (2002). Self-management approaches for people with chronic conditions: a review. Patient Educ Couns.

[13] Liddy C, Blazkho V, Mill K (2014). Challenges of self-management when living with multiple chronic conditions: systematic review of the qualitative literature. Can Fam Physician.

[14] Gobeil-Lavoie AP, Chouinard MC, Danish A, Hudon C, et al. BMJ Open. 2019;9(5). 10.1136/bmjopen-2018-028344 PMC6538095 31129599

[15] Vidan MT, Martin Sanchez FJ, Sanchez E, Ortiz FJ, Serra-Rexach JA, Martinez-Selles M (2019). Most elderly patients hospitalized for heart failure lack the abilities needed to perform the tasks required for self-care: impact on outcomes. Eur J Heart Fail.

[16] Bratzke LC, Muehrer RJ, Kehl KA, Lee KS, Ward EC, Kwekkeboom KL (2015). Self-management priority setting and decision-making in adults with multimorbidity: a narrative review of literature. Int J Nurs Stud.

[17] Hughes LD, McMurdo ME, Guthrie B (2013). Guidelines for people not for diseases: the challenges of applying UK clinical guidelines to people with multimorbidity. Age Ageing.

[18] Coventry PA, Fisher L, Kenning C, Bee P, Bower P (2014). Capacity, responsibility, and motivation: a critical qualitative evaluation of patient and practitioner views about barriers to self-management in people with multimorbidity. BMC Health Serv Res.

[19] Slightam CA, Brandt K, Jenchura EC, Lewis ET, Asch SM, Zulman DM (2018). “I had to change so much in my life to live with my new limitations”: multimorbid patients' descriptions of their most bothersome chronic conditions. Chronic Illn.

[20] Kernick D, Chew-Graham CA, O'Flynn N (2017). Clinical assessment and management of multimorbidity: NICE guideline. Br J Gen Pract.

[21] Cramm JM, Nieboer AP (2015). Chronically ill patients' self-management abilities to maintain overall well-being: what is needed to take the next step in the primary care setting?. BMC Fam Pract.

[22] Sinnott C, Mc Hugh S, Browne J, Bradley C. GPs' perspectives on the management of patients with multimorbidity: systematic review and synthesis of qualitative research. BMJ Open. 2013;3(9). 10.1136/bmjopen-2013-003610 PMC3773648 24038011

[23] Smith SM, Wallace E, O'Dowd T, Fortin M. Interventions for improving outcomes in patients with multimorbidity in primary care and community settings. Cochrane Database Syst Rev. 2016;3. 10.1002/14651858.CD006560.pub3 PMC6703144 26976529

[24] Salisbury C, Man MS, Bower P, Guthrie B, Chaplin K, Gaunt DM (2018). Management of multimorbidity using a patient-centred care model: a pragmatic cluster-randomised trial of the 3D approach. Lancet..

[25] Mercer SW, Fitzpatrick B, Guthrie B, Fenwick E, Grieve E, Lawson K (2016). The CARE plus study - a whole-system intervention to improve quality of life of primary care patients with multimorbidity in areas of high socioeconomic deprivation: exploratory cluster randomised controlled trial and cost-utility analysis. BMC Med.

[26] Zulman DM, Jenchura EC, Cohen DM, Lewis ET, Houston TK, Asch SM (2015). How can eHealth technology address challenges related to multimorbidity? Perspectives from patients with multiple chronic conditions. J Gen Intern Med.

[27] Pariser P, Pham TT, Brown JB, Stewart M, Charles J. Connecting People With Multimorbidity to Interprofessional Teams Using Telemedicine. Ann Fam Med. 2019;17(Suppl 1):S57-62. 10.1370/afm.2379 PMC6827667 31405877

[28] Mangin D, Parascandalo J, Khudoyarova O, Agarwal G, Bismah V, Orr S. Multimorbidity, eHealth and implications for equity: a cross-sectional survey of patient perspectives on eHealth. BMJ Open. 2019;9(2). 10.1136/bmjopen-2018-023731 PMC6377536 30760515

[29] Runz-Jorgensen SM, Schiotz ML, Christensen U (2017). Perceived value of eHealth among people living with multimorbidity: a qualitative study. J Comorb.

[30] Öberg U, Orre CJ, Isaksson U, Schimmer R, Larsson H, Hornsten A (2018). Swedish primary healthcare nurses' perceptions of using digital eHealth services in support of patient self-management. Scand J Caring Sci.

[31] Davis MM, Freeman M, Kaye J, Vuckovic N, Buckley DI (2014). A systematic review of clinician and staff views on the acceptability of incorporating remote monitoring technology into primary care. Telemed J E Health.

[32] Elbert NJ, van Os-Medendorp H, van Renselaar W, Ekeland AG, Hakkaart-van Roijen L, Raat H, et al. Effectiveness and cost-effectiveness of ehealth interventions in somatic diseases: a systematic review of systematic reviews and meta-analyses. J Med Internet Res. 2014;16(4). 10.2196/jmir.2790 PMC4019777 24739471

[33] Morse JM, Cheek J (2014). Making room for qualitatively-driven mixed-method research. Qual Health Res.

[34] Morse JM (2010). Simultaneous and Sequential Qualitative Mixed Method Designs.

[35] Flink M, Lindblad M, Frykholm O, Kneck Å, Nilsen P, Årestedt K, et al. The Supporting Patient Activation in Transition to Home (sPATH) Intervention: A Study Protocol of a Randomised Controlled Trial Using Motivational Interviewing to Decrease Re-Hospitalisation for Patients With COPD or Heart Failure. BMJ open. 2017;7(7). 10.1136/bmjopen-2016-014178 PMC5734357 28698319

[36] Krueger RA, Casey MA (2009). Focus groups: a practical guide for applied research.

[37] Graneheim UH, Lundman B (2004). Qualitative content analysis in nursing research: concepts, procedures and measures to achieve trustworthiness. Nurse Educ Today.

[38] Graneheim UH, Lindgren BM, Lundman B (2017). Methodological challenges in qualitative content analysis: a discussion paper. Nurse Educ Today.

[39] Krippendorf K (2004). Content analysis: an introduction to its methodology.

[40] Stewart M (2001). Towards a global definition of patient centred care. BMJ..

[41] Pereira Gray DJ, Sidaway-Lee K, White E, Thorne A, Evans PH. Continuity of care with doctors-a matter of life and death? A systematic review of continuity of care and mortality. BMJ Open. 2018;8(6). 10.1136/bmjopen-2017-021161 PMC6042583 29959146

[42] Engström S (2019). Personal physician continuity in primary care associated with fewer emergency room visits. Läkartidningen.

[43] Larsen JH, Risør O, Putnam S (1997). P-R-A-C-T-I-C-A-L: a step-by-step model for conducting the consultation in general practice. Fam Pract.

[44] Kandelaki K, Marrone G, Lundborg CS, Schmidt I, Bjorkman I. Patient-centredness as a quality domain in Swedish healthcare: results from the first national surveys in different Swedish healthcare settings. BMJ Open. 2016;6(1). 10.1136/bmjopen-2015-009056 PMC4716147 26747031

[45] Muth C, van den Akker M, Blom JW, Mallen CD, Rochon J, Schellevis FG (2014). The Ariadne principles: how to handle multimorbidity in primary care consultations. BMC Med.

[46] Coulter A, Entwistle VA, Eccles A, Ryan S, Shepperd S, Perera R. Personalised care planning for adults with chronic or long-term health conditions. Cochrane Database Syst Rev. 2015;(3):Cd010523. 10.1002/14651858.CD010523.pub2. 10.1002/14651858.CD010523.pub2 PMC6486144 25733495

[47] Kuipers SJ, Nieboer AP, Cramm JM (2020). Views of patients with multi-morbidity on what is important for patient-centered care in the primary care setting. BMC Fam Pract.

[48] Bodenheimer T, Lorig K, Holman H, Grumbach K (2002). Patient self-management of chronic disease in primary care. JAMA..

[49] Mudge S, Kayes N, McPherson K. Who is in control? Clinicians' view on their role in self-management approaches: a qualitative metasynthesis. BMJ Open. 2015;5(5). 10.1136/bmjopen-2014-007413 PMC4431068 25943372

[50] Flink M, Brandberg C, Ekstedt M (2019). Why patients decline participation in an intervention to reduce re-hospitalization through patient activation: whom are we missing?. Trials..

