# Supplementary material for: “Standing on common ground” - a qualitative study of self-management support for patients with multimorbidity in primary health care
Source: BMC Fam Pract. 2020 Nov 17;21:233. doi: 10.1186/s12875-020-01290-y (PMC7670978; doi:10.1186/s12875-020-01290-y)
Supplement: Supplementary file 1 — Additional file 1. Examples of categories, subcategories, and codes/quotations color-coded by origin: black for physicians, green for RNs, blue for patients, and red for FCs. [file 12875_2020_1290_MOESM1_ESM.docx]

## Additional file. Examples of categories, subcategories, and codes/quotations color-coded by origin: black for physicians, green for RNs, blue for patients, and red for FCs.

| **Categories** | **Subcategories** | **Codes/quotations** |
| --- | --- | --- |
| Individualized support and patient-professional relationships | Individualized care considers the patient’s agenda and self-management ability | - “They’re more interested in having the skin lesion or the hip examined than discussing their chronic illness.” - Considers patients’ other diseases when goals are set. - “Regarding diabetes and hypertension, I set goals that are measurable.” - Adjusts medications and motivates the patients on an individual basis. - Asks the patients what support they need. - “I prefer being with my children and grandchildren not thinking so much about diseases.” |
|  | Trustful relationships enable self-management support | - “We build a relationship over time that enables the motivational work.” - “I prefer a doctor who knows me to a new one, even though he or she has read my medical chart.” - “I feel that the PHC center regards me as a hypochondriac.” - “Then I believe I should have a proper review of my heart.” - Embarrassing to switch doctors. - “It’s hard to speak up.” |
|  | Support for self-management is more than information | - Patients’ concerns bring the patients to the doctor. - Uncertain how the PHC center can help me. I wish for more people around me. |
| Professionals as knowledge translators to help patients learn self-management skills | Knowledge affects self-management abilities and decreases anxiety | - “Normally I don’t set health goals with my patients if they’re too sick.” - With medical knowledge, the physician can help patients interpret their symptoms and explain the medication. - “I explain what happened at the hospital.” - “I wonder why my body hurts and why cortisone helps.” - “I feel unsure about what symptoms are associated with my chronic heart failure.” - The patient has difficulty separating stomach ache from pain from the heart. - The patient lacks computer experience. - Knowledge enables patient understanding and ability to handle preventive medication. - The family caregiver can help when it is hard for the patient to understand the information. - The patient connects his fatigue with chronic heart failure. - “I increase my medication to decrease the swelling in my feet.” - The symptoms you understand feel less worrying than the ones you cannot interpret. - “The diabetes doesn’t worry me that much. It’s caused by cortisone.” - Anxiety is worse than having a myocardial infarction. |
|  | Pedagogical strengths and weaknesses among health care professionals | - Forgets to ask what the patient remembers from the consultation. - The physician uses pictures to explain. - The physician clarifies the list of medications. - Don’t get explanations for his symptoms. - “The GP doesn’t bother to listen to my own explanations.” |
|  | Self-monitoring enables self-management | - “You walk more when you have a pedometer, that’s how you work.” - “During the summer, I registered my symptoms differently when it was hot, and I felt shortness of breath.” - “I believe it’s possible to be able to check my health measurements instead of having to go the PHC center.” - “I’ve noticed that my blood pressure improves if I increase my medication for a couple of days.” - “I’ve learned to understand my symptoms better thanks to registering them.” - An advantage of digital measurements is that you see deterioration immediately. |
| Managing multimorbidity and coordinating care in a system focused on single diseases | PHC’s role in coordinating care in a fragmented system | - Patients receive information from many sources. - When RNs specialize in various diseases, it results in multiple visits to different RNs in PHC. - “It would be a dream to have a system [electronic record] that had an overview of all diseases.” - Other specialists speak degradingly about PHC physicians. - Do not know if the doctor at the hospital communicates with the PHC center. - “I wish the home care service could also take part in the home monitoring.” - “I coordinate the patient’s care between the hospital, home care, rehab, and care managers.” - The older patients need support from home care service. |
|  | Accessibility to health care and time for patients with multimorbidity enhances self-management support | - It is complex to assess patients with multimorbidity and polypharmacy. - Hard to evaluate blood samples in patients with multimorbidity. - “I had both swollen legs and itching from the new medication.” - “It’s harder to keep my weight down with insulin.” - “My hips are worn out, which makes it harder for me to move.” - “I have a difficulty affecting my blood sugar since it’s hard for me to exercise.” - When drugs change colors, it makes it hard to know which medication is which. - People want to see their physician more than ever. - Simple health problems take too much time. - There can be two months between visits to the PHC center, so you have to solve as much as possible at one visit. - Lack of physician appointments results in a higher number of acute visits when patients get worse. - There is insufficient time to investigate patients with multimorbidity. - Even though it’s a visit to check blood pressure or diabetes, patients bring up other concerns. - “I prefer being able to contact my physician via telephone.” - The tablet makes it easier to access the PHC center. |
| Shifting roles and differing views of responsibility for self-management | When PHC professionals are in control, it increases adherence and patient safety and reduces anxiety | - “I assume no one does what you tell them to do.” - “I ask the RN to contact the patient if I see that the patient’s disease gets worse.” - “I find solutions to follow up symptoms that can be potentially severe.” - Going through what patients have written on their notes can reveal severe symptoms. - Patients need more than just support for self-management when they are so sick. - “I find that the PHC center contacts me when needed.” - “It feels safe that someone is monitoring me.” - Normally health care doesn’t trust the patient’s own judgement. - “I’ve seen that the RNs monitor the health measurements.” - “The best part of the monitoring is that the PHC is able to respond to deterioration in time.” - The FC notices changes in the patient’s condition by being there every day. |
|  | Empowering patients enables a shift in responsibility | - Patients whose disease is stable can see an RN. - Patients believe that if the blood tests are fine, they do not have to bother about their chronic illness. - Trivial everyday problems can hinder lifestyle change. - “I take care of the sickest patients, while the assistant RN can take of less complicated tasks.” - Patients become more involved in their self-management by monitoring health data. - Self-management is when patients self-monitor, take more responsibility for their diseases, and acquire more knowledge. - If there is no motivation it’s hard to stop smoking. - “I believe that I’ve been made responsible for doing certain checks that PHC did before.” - “Before I started monitoring my health data, there was no structure.” - “I appreciate the routine of monitoring my health data.” - “I contact PHC if I need to renew prescriptions.” - “I wouldn’t contact PHC more than I do today.” |
